# Exercise *in vivo* marks human myotubes *in vitro*: Training-induced increase in lipid metabolism

**DOI:** 10.1371/journal.pone.0175441

**Published:** 2017-04-12

**Authors:** Jenny Lund, Arild C. Rustan, Nils G. Løvsletten, Jonathan M. Mudry, Torgrim M. Langleite, Yuan Z. Feng, Camilla Stensrud, Mari G. Brubak, Christian A. Drevon, Kåre I. Birkeland, Kristoffer J. Kolnes, Egil I. Johansen, Daniel S. Tangen, Hans K. Stadheim, Hanne L. Gulseth, Anna Krook, Eili T. Kase, Jørgen Jensen, G. Hege Thoresen

**Affiliations:** 1 Department of Pharmaceutical Biosciences, School of Pharmacy, University of Oslo, Oslo, Norway; 2 Integrative Physiology, Department of Physiology and Pharmacology, Karolinska Institutet, Stockholm, Sweden; 3 Department of Nutrition, Institute of Basic Medical Sciences, University of Oslo, Oslo, Norway; 4 Department of Endocrinology, Morbid Obesity and Preventive Medicine, Oslo, University Hospital and Institute of Clinical Medicine, University of Oslo, Oslo, Norway; 5 Department of Physical Performance, Norwegian School of Sport Sciences, Oslo, Norway; 6 Department of Pharmacology, Institute of Clinical Medicine, University of Oslo, Oslo, Norway; Tohoku University, JAPAN

## Abstract

**Background and aims:**

Physical activity has preventive as well as therapeutic benefits for overweight subjects. In this study we aimed to examine effects of *in vivo* exercise on *in vitro* metabolic adaptations by studying energy metabolism in cultured myotubes isolated from biopsies taken before and after 12 weeks of extensive endurance and strength training, from healthy sedentary normal weight and overweight men.

**Methods:**

Healthy sedentary men, aged 40–62 years, with normal weight (body mass index (BMI) < 25 kg/m^2^) or overweight (BMI ≥ 25 kg/m^2^) were included. Fatty acid and glucose metabolism were studied in myotubes using [^14^C]oleic acid and [^14^C]glucose, respectively. Gene and protein expressions, as well as DNA methylation were measured for selected genes.

**Results:**

The 12-week training intervention improved endurance, strength and insulin sensitivity *in vivo*, and reduced the participants’ body weight. Biopsy-derived cultured human myotubes after exercise showed increased total cellular oleic acid uptake (30%), oxidation (46%) and lipid accumulation (34%), as well as increased fractional glucose oxidation (14%) compared to cultures established prior to exercise. Most of these exercise-induced increases were significant in the overweight group, whereas the normal weight group showed no change in oleic acid or glucose metabolism.

**Conclusions:**

12 weeks of combined endurance and strength training promoted increased lipid and glucose metabolism in biopsy-derived cultured human myotubes, showing that training *in vivo* are able to induce changes in human myotubes that are discernible *in vitro*.

## Introduction

Physical activity has preventive as well as therapeutic benefits for metabolic diseases associated with insulin resistance such as obesity and type 2 diabetes mellitus (T2D) [1, 2]. In addition to increased physical activity, dietary changes and weight loss are important lifestyle changes for prevention as well as treatment of T2D [2], as increased body mass index (BMI) is strongly associated with the prevalence of metabolic diseases [3, 4], and most type 2 diabetics are overweight or obese [5]. Physical activity is known to improve insulin sensitivity and glucose homeostasis and to increase fatty acid oxidation in skeletal muscle [6–8], as well as to reduce blood pressure and beneficially influence plasma lipoproteins [9].

Skeletal muscle is the largest glucose-consuming organ in the body and accounts for more than 80% of the insulin-stimulated glucose disposal [10]. Skeletal muscle is also the primary site for insulin resistance [11]. Also with regard to fatty acid metabolism, skeletal muscle is quantitatively the most dominant tissue during exercise [7]. Satellite cells [12] are dormant cells in mature skeletal muscle *in vivo*, but are activated in response to stress, *e*.*g*. muscle growth [13], and may be activated in culture to proliferating myoblasts and differentiated into multinucleated myotubes. Epigenetic changes such as DNA methylation of key regulatory genes has been proposed as one of several molecular mechanisms to explain the beneficial effects of lifestyle changes, as both diet and exercise can influence DNA methylation [14, 15]. Several studies indicate that cultured myotubes retain the *in vivo* characteristics (see *e*.*g*. [11, 16–20]), and although the precise mechanisms are not known, epigenetic changes may be involved (discussed in [21]). Thus, cultured human myotubes may represent an *ex vivo* model system for intact human skeletal muscle [19].

Most studies on the effect of exercise on metabolic diseases have been performed *in vivo* [22, 23] or directly on muscle biopsies [24, 25]. However, a study on obese donors revealed that enhanced glucose metabolism noted *in vivo* following 8 weeks aerobic exercise, was preserved in cultured primary myotubes [16]. To further explore the effects of *in vivo* exercise on *in vitro* metabolic adaptations, we studied different aspects of energy metabolism in cultured myotubes established from biopsies from healthy sedentary normal weight and overweight men. Biopsies were obtained before and after 12 weeks of physical training, consisting of both endurance and strength exercises.

## Materials and methods

### Materials

Materials are reported in Table 1.

**Table 1:** List of materials and respective producers.

Material	Producer
Nunc^™^ Cell Culture Treated Flasks with Filter Caps	ThermoFisher Scientific (Roskilde, Denmark)
Nunc^™^ 96-MicroWell^™^ plates	
Pierce^™^ BCA Protein Assay Kit	
SuperSignal West Femto Maximum Sensitivity Substrate	
O´GeneRuler 100 bp DNA ladder	
Antibody against phosphorylated IRS1 at Tyr612 (#44-816G)	
Primers for TaqMan PCR	
DMEM-Glutamax^™^ low glucose with sodium pyruvate	Gibco Invitrogen (Gibco, Life Technologies, Paisley, UK)
FBS	
Trypsin-EDTA	
Penicillin-streptomycin (10000 IE/ml)	
Amphotericin B	
DPBS (without Mg^2+^ and Ca^2+^)	
Ultroser G	Pall (Cergy-Saint-Christophe, France)
Insulin (Actrapid^®^ Penfill^®^ 100 IE/ml)	Novo Nordisk (Bagsvaerd, Denmark)
Trypan blue 0.4% solution	Sigma-Aldrich (St. Louis, MO, US)
DMSO	
L-glutamine	
BSA (essentially fatty-acid free)	
L-carnitine	
D-glucose	
Oleic acid (OA, 18:1, n-9)	
HEPES	
Glycogen	
β-mercaptoethanol	
Primers for PyroMark PCR and pyrosequencing	
96-well Corning^®^ CellBIND^®^ tissue culture plates	Corning (Schiphol-Rijk, the Netherlands)
VWR^®^ Grade 703 Blotting Paper	VWR (Poole, UK)
[1-^14^C]oleic acid (2.083 GBq/mmol)	PerkinElmer NEN^®^ (Boston, MA, US)
D-[^14^C(U)]glucose (9.25 GBq/mmol)	
OptiPhase Supermix	PerkinElmer (Shelton, CT, US)
96-well Isoplate^®^	
Unifilter^®^-96 GF/B	
TopSeal^®^-A transparent film	
MultiScreen^®^ HTS hydrophobic filter plates with high-protein binding Immobilon-P membrane	Millipore (Billerica, MA, US)
GelRed^™^ Nucleic Acid Gel Stain 10000X in water	Biotium (Hayward, CA, US)
Clarity^™^ Western ECL Substrate	BioRad (Copenhagen, Denmark)
Tris/glycine buffer	
Tris/glycine/SDS buffer	
SDS	
Tween 20	
Bromophenol blue	
Goat Anti-Rabbit IgG (H+L)-HRP Conjugate (#170–6515)	
Goat Anti-Mouse IgG (H+L)-HRP Conjugate (#170–6516)	
Mini-Protean^®^ TGX^™^ gels (4–20%)	
Bio-Rad Protein Assay Dye Reagent Concentrate	
Glycerol	Merck (Darmstadt, Germany)
Tris-HCl	
Amersham^™^ Protran^™^ Premium 0.45 μm NC Nitrocellulose Blotting Membrane	Amersham^™^ (GE Healthcare, Esbjerg, Denmark)
Antibodies against human total and phosphorylated Akt at Ser473 (#9272 and #9271S, respectively)	Cell Signaling Technology Inc. (Beverly, MA, US)
Antibodies against total and phosphorylated TBC1D4 at Thr642 (#2670 and #4288, respectively)	
Antibodies against total and phosphorylated AMPKα at Thr172 (#2531 and #2532, respectively)	
Antibody against total IRS1 (#3407)	
Antibody against MHCIIa (#3403S)	
Antibody against α-tubulin (#2144)	
Antibody against MHCI (#MAB1628)	Millipore (Temecula, CA, US)
Antibodies against human total OXPHOS (#110411)	Abcam (Cambridge, UK)
RNeasy Mini Kit	QIAGEN (Venlo, the Netherlands)
DNeasy Blood & Tissue Kit	
EpiTect Fast DNA Bisulfite Conversion Kit	
PyroMark^®^ PCR Kit	
PyroMark^®^ Q24 Advanced CpG Reagents	
PyroMark^®^ Q24 Plate	
PyroMark^®^ Wash Buffer	
PyroMark^®^ Denaturation Buffer	
PyroMark^®^ Q24 Cartridge	
Streptavidin Sepharose^®^ High Performance beads	GE Healthcare Life Sciences (Little Chalfont, UK)
TaqMan reverse transcription kit reagents	Applied Biosystems (Warrington, UK)
MicroAmp^®^ Optical 96-well Reaction Plate	
MicroAmp^®^ Optical Adhesive Film	
High-Capacity cDNA Reverse Transcription Kit	
Power SYBR^®^ Green PCR Master Mix	

### Ethics statement

The biopsies were obtained after informed written consent and approval by the Regional Committee for Medical and Health Research Ethics North, Tromsø, Norway (reference number: 2011/882). The research performed in this study was approved, as part of a larger project: Skeletal Muscles, Myokines and Glucose Metabolism (MyoGlu) [26]. The study adhered to the Declaration of Helsinki, and it was registered with the US National Library of Medicine Clinical Trials registry (NCT01803568).

### Donor characteristics

The biopsies were obtained from 18 volunteer men before and after participating in a 12-week exercise intervention program at the Norwegian School of Sports Sciences, Oslo, Norway. The biopsies were taken 2 hours after an acute exercise test [26]. To take part in the study the participants had to be sedentary men (not regularly exercising more than once a week), 40 to 62 years old, non-smokers and of Nordic ethnicity. Blood samples were analyzed at Oslo University Hospital during clamp measurements or at Fürst Laboratories (Oslo, Norway). Prior to a euglycemic hyperinsulinemic clamp, body composition by bioelectric impedance analysis was performed with Tanita Body Composition Analyzer BC-418 MA. Both the clamp and bioimpedance measurements were performed under strict criteria, *e*.*g*. fasting from the night before, no alcohol or exercise the last 48 hours and empty bladder before bioimpedance analysis.

The group was further divided in two groups, normal weight and overweight, *i*.*e*. below and above the World Health Organization’s lower limit for overweight (BMI 25 kg/m^2^), respectively, for all analyses except glycogen synthesis and DNA methylation experiments where only a subset of the donors were examined (n < 3 in the normal weight group).

### Exercise training

The training program was performed at the Norwegian School of Sport Sciences. Each participant exercised 4 times per week for 12 weeks, both endurance sessions twice weekly and strength training sessions twice weekly. Endurance sessions consisted of interval-based cycling, and strength training sessions consisted of 3 sets of 8 exercises (leg press, arm press, chest press, cable pull-down, leg curls, crunches, seated rowing, and a back exercise). All sessions were supervised by one instructor for two participants. Each session, whether endurance or strength training, lasted about 60 min, excluding 10–20 min aerobic warm-up. The endurance exercise was performed with two different intervals; one of the sessions was performed at 7 min intervals, whereas the other session was performed at 2 min intervals. Compliance to the exercise intervention was equally good in the two BMI groups [26].

Maximal strength was tested before and after the exercise intervention in maximal leg press, cable pull-down, and breast press, whereas endurance capacity before and after the exercise intervention was evaluated as maximal oxygen uptake (VO_2max_) after 45 min cycling at 70% of estimated VO_2max_. Each participant followed a standardized warm-up before testing.

Dietary intakes were registered by a food frequency questionnaire [27] before and after the exercise intervention. There was no significant change in intake of energy-providing nutrients during the study [28].

### Culturing of human myotubes

Multinucleated human myotubes were established by activation and proliferation of satellite cells isolated from *musculus vastus lateralis* from 7 sedentary normal weight men and from 11 sedentary overweight men. This was based on the method of Henry *et al*. [29] and modified according to Gaster *et al*. [30, 31]. For proliferation of myoblasts a DMEM-Glutamax^™^ (5.5 mmol/l glucose) medium supplemented with 2% FBS and 2% Ultroser G were used. At approximately 80% confluence the medium was changed to DMEM-Glutamax^™^ (5.5 mmol/l glucose) supplemented with 2% FBS and 25 pmol/l insulin to initiate differentiation into multinucleated myotubes. The cells were allowed to differentiate for 7 days; no difference in cell differentiation could be detected based on protein expressions of MHCI and MHCIIa (S1 Fig), and by visual examination in the microscope. During the culturing process the muscle cells were incubated in a humidified 5% CO_2_ atmosphere at 37°C, and medium was changed every 2–3 days. Experiments were performed on cells from passage number 2 to 4. For each experiment and within each donor, *i*.*e*. before and after exercise, the passage number remained constant. Isolation of satellite cells from all biopsies was performed at the same location and by the same trained researchers. Skeletal muscle cultures have previously been checked for the adipocyte marker fatty acid binding protein (FABP) 4 to ensure a homogenous skeletal muscle cell-population. All cell cultures were visually checked for fibroblast content throughout proliferation.

### Fatty acid and glucose metabolism

Skeletal muscle cells (7000 cells/well) were cultured on 96-well CellBIND^®^ microplates. [1-^14^C]oleic acid (18.5 kBq/ml), 20, 100 or 400 μmol/l, or D-[^14^C(U)]glucose (21.46 kBq/ml), 200 μmol/l, were given during 4 h CO_2_ trapping as previously described [32]. In brief, a 96-well UniFilter^®^-96 GF/B microplate was mounted on top of the CellBIND^®^ plate and CO_2_ production was measured in DPBS medium with 10 mmol/l HEPES and 1 mmol/l L-carnitine adjusted to pH 7.2–7.3. CO_2_ production and cell-associated (CA) radioactivity were assessed using a 2450 MicroBeta^2^ scintillation counter (PerkinElmer). The sum of ^14^CO_2_ and remaining CA radioactivity was taken as a measurement of total cellular uptake of substrate: CO_2_+CA. Fractional complete oxidation was calculated as: $\frac{{\text{CO}}_{2}}{{\text{CO}}_{2}+\text{CA}}$. Fractional oxidation gives a picture of what proportion of the substrate taken up that is oxidized and may or may not correlate to oxidation calculated per amount protein (or cells), depending on the regulation of the different processes: uptake and oxidation. Thus, an increased fractional oxidation indicates that substrate oxidation is increased relative to the substrate uptake. Protein levels in the lysate were measured by the Bio-Rad protein assay using a VICTOR^™^ *X*4 Multilabel Plate Reader (PerkinElmer).

### Determination of lipid accumulation

To study whether an alteration of the radiolabeled oleic acid occurs and if it is incorporated into complex lipids within the myotubes, lipid filtration was performed. Lysate from the fatty acid oxidation assays were filtrated through hydrophobic MultiScreen^®^ HTS filter plates. The total amount of complex lipids in the cell lysates was determined by liquid scintillation. Lipid filtration has previously been evaluated against thin layer chromatography and found equal in describing levels of total complex lipids in a cell lysate [33].

### Glycogen synthesis

Myotubes were exposed to serum-free DMEM supplemented with [^14^C(U)]glucose (18.5 kBq/ml, 0.17 mmol/l) and 0.5 mmol/l unlabeled glucose, in presence or absence of 100 nmol/l insulin (Actrapid^®^ Penfill 100 IE/ml) for 3 h to measure glycogen synthesis. In preliminary unpublished studies, we have seen a defective insulin-stimulated glycogen synthesis at all concentrations of insulin, ranging from 1 nmol/l to 100 nmol/l. Thus, we decided to use 100 nmol/l insulin to reach maximal insulin stimulation in all experiments. The cells were washed twice with PBS and harvested in 1 mol/l KOH. Protein content was determined by use of the Pierce BCA Protein Assay Kit, before 20 mg/ml glycogen and more KOH (final concentration 4 mol/l) were added to the samples. Then, [^14^C(U)]glucose incorporated into glycogen was measured as previously described [34].

### Immunoblotting

Myotubes were incubated with or without 100 nmol/l insulin for 15 min before the cells were harvested in Laemmli buffer (0.5 mol/l Tris-HCl, 10% SDS, 20% glycerol, 10% β-mercaptoethanol, and 5% bromophenol blue). The proteins were electrophoretically separated on 4–20% Mini-Protean^®^ TGX^™^ gels with Tris/glycine buffer (pH 8.3) followed by blotting to nitrocellulose membrane and incubation with antibodies for total Akt kinase and Akt phosphorylated at Ser473, total insulin receptor substrate (IRS) 1 and IRS1 phosphorylated at Tyr612, total TBC1 domain family member 4 (TBC1D4, also known as Akt substrate of 160 kDa, AS160) and TBC1D4 phosphorylated at Thr642, total AMP-activated protein kinase (AMPK) and AMPK phosphorylated at Thr172, MHCI, MHCIIa, total oxidative phosphorylation (OXPHOS) complexes, and α-tubulin. Immunoreactive bands were visualized with enhanced chemiluminescence (Chemidoc XRS, BioRad, Copenhagen, Denmark) and quantified with Image Lab (version 4.0) software. Myotubes from 10 donors were used for the pTBC1D4/total TBC1D4, MHCI, MHCIIa, and OXPHOS analyses, whereas myotubes from 9 donors were used for the pAkt/total Akt, pIRS1/total IRS1 and pAMPKα/total AMPKα analyses. All samples were derived at the same time and processed in parallel. Expression levels were normalized to one sample used as loading control. Expressions of MHCI, MHCIIa, OXPHOS complex V, and total IRS1 were further normalized to the endogenous control α-tubulin.

### RNA isolation and analysis of gene expression by qPCR

Total RNA was isolated from myotubes using RNeasy Mini Kit according to the supplier´s protocol. RNA was reversely transcribed with a High-Capacity cDNA Reverse Transcription Kit and TaqMan Reverse Transcription Reagents using a PerkinElmer 2720 Thermal Cycler (25°C for 10 min, 37°C for 80 min, 85°C for 5 min). Primers were designed using Primer Express^®^ (Applied Biosystems). qPCR was performed using a StepOnePlus Real-Time PCR system (Applied Biosystems). Target genes were quantified in duplicates carried out in a 25 μl reaction volume according to the supplier´s protocol. All assays were run for 44 cycles (95°C for 15 s followed by 60°C for 60 s). Expression levels were normalized to the average of the housekeeping gene *GAPDH* (acc.no. NM002046). The housekeeping gene large ribosomal protein P0 (*RPLP0*, acc.no. M17885) was also analyzed; there were no differences between normalizing for *GAPDH* or *RPLP0*. The following forward and reverse primers were used at concentration of 30 μmol/l, *GAPDH*; *RPLP0*; pyruvate dehydrogenase kinase, isoenzyme 4 (*PDK4*, acc.no. BC040239); angiopoietin-like 4 (*ANGPTL4*, acc.no. NM139314); carnitine palmitoyltransferase 1A (*CPT1A*, acc.no. L39211); perilipin 2 (*PLIN2*, acc.no. NM001122); fatty acid translocase (*CD36*, acc.no. L06850); cytochrome c-1 (*CYC1*, acc.no. NM001916); peroxisome proliferator-activated receptor gamma, coactivator 1 alpha (*PPARGC1A*, acc.no. NM013261.3); peroxisome proliferator-activated receptor delta (*PPARD*, acc.no. BC002715); *IRS1* (acc.no. NM_005544.2).

### DNA methylation measurement

gDNA was extracted from myotubes using DNeasy Blood & Tissue Kit. A concentration of ≥20 ng/μl was used. The gDNA was bisulfite treated using EpiTect Fast DNA Bisulfite Kit. Forward, reverse and sequencing primers for *PDK4*, *PPARGC1A*, *PPARD*, mitochondrial transcription factor A (*TFAM*), and *IRS1* were designed using PyroMark AssayDesign 2.0 (QIAGEN, Venlo, the Netherlands). We tested 3 CpGs in the promoter region of *PKD4* (chr7:95,226,252–95,226,322), 2 CpGs in the promoter of *PPARGC1A* (chr4:23,891,715–23,891,726), 4 CpGs in the promoter of *PPARD* (chr6:35,309,819–35,309,931), 8 CpGs in the promoter of *TFAM* (chr10:60,144,788–60,144,828), and 3 CpGs in the first exon of *IRS1* (chr2:227,661,201–227,661,293). For each primer-set, bisulfite-treated DNA was amplified by PCR using PyroMark PCR Kit and MyCycler Thermal Cycler (BioRad, Copenhagen, Denmark). The reaction was visualized by gel electrophoresis to check if it was the right product according to the size and if it was well amplified with no secondary product. The reaction was optimized if necessary. DNA methylation for each region of interest was measured by pyrosequencing using QIAGEN PyroMark Q24.

### Presentation of data and statistics

Data are presented as means ± SEM. The value *n* represents the number of different donors; each *in vitro* experiment with at least duplicate observations. For immunoblotting, results for normal weight group before exercise was set to 100%, and for experiments with insulin-stimulation, basal before exercise was set to 100%. Statistical analyses were performed using GraphPad Prism 6.0c for Mac (GraphPad Software, Inc., La Jolla, CA, US) or SPSS version 22 (IBM^®^ SPSS^®^ Statistics for Macintosh, Armonk, NY, US). Linear mixed-model analysis was used to compare differences between conditions with within-donor variation and simultaneously compare differences between groups with between-donor variation. The linear mixed-model analysis includes all observations in the statistical analyses and takes into account that not all observations are independent. Paired t test was used within groups, whereas unpaired t test with equal standard deviation was used to evaluate effects between groups. Correlation studies were performed with Pearson’s test and are presented as Pearson’s correlation coefficient (r). A *p*-value < 0.05 was considered significant.

## Results

### Donor characteristics

Donor characteristics pre- and post-training are presented in Table 2. After 12 weeks of exercise both normal weight and overweight donor groups significantly increased maximal strength and insulin sensitivity measured as the glucose infusion rate (GIR). Only the normal weight group significantly reduced percentage body fat (overweight: *p* = 0.07) after the exercise intervention, whereas only the overweight group significantly increased VO_2max_ (normal weight: *p* = 0.053) and reduced body weight and BMI. Visceral fat area also tended to be smaller after the exercise intervention in the overweight group (*p* = 0.07).

**Table 2:** Clinical and biochemical variables in normal weight (BMI < 25 kg/m^2^) and overweight men (BMI ≥ 25 kg/m^2^) at baseline (pre-training) and after 12 weeks of extensive endurance and strength training (post-training).

	Pre-training all donors	Post-training all donors	Pre-training normal weight	Post-training normal weight	Pre-training overweight	Post-training overweight
n	18	18 (17^†^)	7	7 (6^†^)	11	11
Age, y	50.4 ± 1.6	-	48.0 ± 2.8	-	51.9 ± 1.8	-
Body weight, kg	88.6 ± 3.2	87.1 ± 3.0*	78.4 ± 3.2	78.1 ± 3.3	95.1 ± 3.6^#^	92.8 ± 3.5*^#^
BMI, kg/m^2^	27.0 ± 0.9	26.6 ± 0.8*	23.3 ± 0.7	23.3 ± 0.6	29.4 ± 0.7^#^	28.7 ± 0.7*^#^
Waist-hip ratio	0.92 ± 0.01	0.91 ± 0.01	0.88 ± 0.01	0.88 ± 0.01	0.95 ± 0.01^#^	0.94 ± 0.01^#^
Fat mass, %	23.2 ± 1.2	22.1 ± 1.2*	18.0 ± 1.0	16.8 ± 0.8*	26.5 ± 0.9^#^	25.4 ± 1.0^#^
Visceral fat area, cm^2^	138.0 ± 12.5	118.4 ± 9.7	101.6 ± 15.0	90.5 ± 5.9	161.1 ± 14.4^#^	136.1 ± 13.0^#^
Fasting glucose, mmol/l	5.6 ± 0.1	5.7 ± 0.1*	5.3 ± 0.2	5.5 ± 0.2	5.7 ± 0.1	5.9 ± 0.1
GIR, mg/kg/min	6.0 ± 0.6	7.8 ± 0.8*	7.7 ± 0.7	9.4 ± 0.9*	5.0 ± 0.7^#^	6.7 ± 1.0*
VO_2max_, ml/kg/min	39.2 ± 1.1	44.1 ± 1.5*	42.5 ± 0.9	47.1 ± 2.2	37.1 ± 1.5^#^	42.3 ± 1.8*
Chest press_max_, kg	67.5 ± 3.6	77.6 ± 4.0*^†^	61.8 ± 4.7	71.7 ± 6.4*^†^	71.1 ± 5.0	80.9 ± 5.1*
Cable pull-down_max_, kg	72.5 ± 3.3	82.4 ± 3.0*^†^	68.9 ± 3.7	78.3 ± 3.4*^†^	74.8 ± 4.9	84.5 ± 4.4*
Leg press_max_, kg	224.9 ± 10.4	249.3 ± 11.7*	192.9 ± 14.6	209.3 ± 13.9*	245.2 ± 10.5^#^	274.8 ± 11.7*^#^

Glucose infusion rate (GIR) measurements were performed with euglycemic hyperinsulinemic clamp analysis; visceral fat area and skeletal muscle mass were based on bioelectrical impedance analysis with Tanita. Values are given as means ± SEM (n = 7 in the normal weight group and n = 11 in the overweight group).

*Statistically significant vs. pre-training (*p* < 0.05, paired t test).

^#^Statistically significant vs. normal weight (*p* < 0.05, unpaired t test with equal SD).

^†^Missing data from one normal weight participant for the post-exercise tests in the two arm exercises (chest press and cable pull-down) due to an arm injury.

BMI, body mass index; VO_2max_, maximal oxygen uptake.

As expected, there were significant differences between the normal weight group and the overweight group both pre- and post-training for body weight, BMI, waist-hip ratio, percentage body fat, visceral fat area, and maximal strength in leg press (Table 2). GIR and VO_2max_ only differed pre-training between the groups.

### Increased fatty acid and glucose metabolism in cultured human myotubes after 12 weeks of exercise

**Fig 1:**
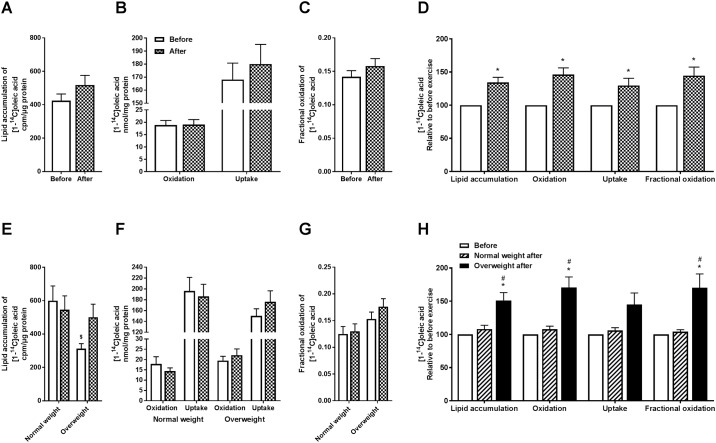
Effects of 12 weeks of exercise on myotube fatty acid metabolism. Satellite cells isolated from biopsies from *m*. *vastus lateralis* before and after 12 weeks of exercise were cultured and differentiated to myotubes. Oxidation, cell-associated (CA) radioactivity and lipid accumulation of [^14^C]oleic acid were measured, and total cellular uptake (CO_2_+CA), oxidation (CO_2_), fractional oxidation ($\frac{{\text{CO}}_{2}}{{\text{CO}}_{2}+\text{CA}}$), and lipid accumulation were determined. **(A)** Lipid accumulation presented as cpm/μg protein. Values are presented as means ± SEM for all participants combined (n = 18). **(B)** Oleic acid oxidation and total cellular uptake presented as nmol/mg protein. Values are presented as means ± SEM for all participants combined (n = 18). **(C)** Fractional oleic acid oxidation. Values are presented as means ± SEM for all participants combined (n = 18). **(D)** Fatty acid metabolism relative to before exercise. Values are presented as means ± SEM for all participants combined (n = 18). **(E)** Lipid accumulation presented as cpm/μg protein in study group when separated by BMI. Values are presented as means ± SEM (n = 7 in the normal weight group and n = 11 in the overweight group). **(F)** Oleic acid oxidation and total cellular uptake presented as nmol/mg protein in study group when separated by BMI. Values are presented as means ± SEM (n = 7 in the normal weight group and n = 11 in the overweight group). **(G)** Fractional oleic acid oxidation in absolute values in study group when separated by BMI. Values are presented as means ± SEM (n = 7 in the normal weight group and n = 11 in the overweight group). **(H)** Fatty acid metabolism relative to before exercise in study group separated by BMI. Values are presented as means ± SEM (n = 7 in the normal weight group and n = 11 in the overweight group). *Statistically significant vs. before exercise (*p* < 0.05, linear mixed-model analysis, SPSS). ^#^Statistically significant vs. normal weight group after exercise (*p* < 0.05, linear mixed-model analysis, SPSS). ^$^Statistically significant vs. normal weight group (*p* < 0.05, linear mixed-model analysis, SPSS).

Fatty acid metabolism in myotubes obtained from biopsies before and after 12 weeks of exercise is presented in Fig 1. Results for all participants combined (n = 18) are shown in Fig 1A–1D, and separated by BMI in Fig 1E–1H. The overall statistically significant exercise-induced increase in total cellular oleic acid uptake was 30%, in oleic acid oxidation 46%, in fractional oxidation 45%, and in lipid accumulation of oleic acid 34% (Fig 1D). When study group was separated by BMI, myotubes from the overweight group showed exercise-induced increase in oleic acid oxidation, fractional oxidation and lipid accumulation by 71%, 70%, and 51%, respectively, after exercise (Fig 1H). Total cellular oleic acid uptake also tended to be increased after the exercise intervention in the overweight group (*p* = 0.08, Fig 1H). There were no statistically significant exercise-induced changes in oleic acid metabolism in myotubes from the normal weight group (Fig 1H). In myotubes established before exercise, lipid accumulation was lower in the overweight group compared to the normal weight group (Fig 1E). Pre-training lipid accumulation correlated significantly positively with GIR (r = 0.47, and *p* = 0.05) and negatively with fasting glucose (r = -0.53 and *p* = 0.03), suggesting a relationship between lipid accumulation and insulin sensitivity (data not shown).

**Fig 2:**
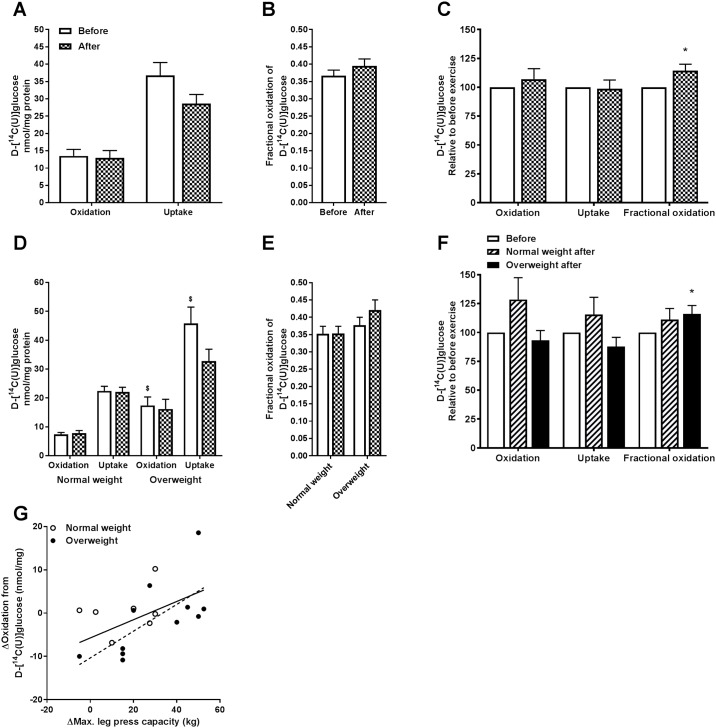
Effects of 12 weeks of exercise on myotube glucose metabolism. Satellite cells isolated from biopsies from *m*. *vastus lateralis* before and after 12 weeks of exercise were cultured and differentiated to myotubes. Oxidation and cell-associated (CA) radioactivity of [^14^C]glucose were measured, and total cellular uptake (CO_2_+CA), oxidation (CO_2_), and fractional oxidation ($\frac{{\text{CO}}_{2}}{{\text{CO}}_{2}+\text{CA}}$) were determined. **(A)** Glucose oxidation and total cellular uptake presented as nmol/mg protein. Values are presented as means ± SEM for all participants combined (n = 18). **(B)** Fractional glucose oxidation. Values are presented as means ± SEM for all participants combined (n = 18). **(C)** Glucose metabolism relative to before exercise. Values are presented as means ± SEM for all participants combined (n = 18). *Statistically significant vs. before exercise (*p* < 0.05, linear mixed-model analysis, SPSS). **(D)** Glucose oxidation and total cellular uptake presented as nmol/mg protein in study group when separated by BMI. Values are presented as means ± SEM (n = 7 in the normal weight group and n = 11 in the overweight group). **(E)** Fractional glucose oxidation in absolute values in study group when separated by BMI. Values are presented as means ± SEM (n = 7 in the normal weight group and n = 11 in the overweight group). **(F)** Glucose metabolism relative to before exercise in study group when separated by BMI. Values are presented as means ± SEM (n = 7 in the normal weight group and n = 11 in the overweight group). *Statistically significant vs. before exercise (*p* < 0.05, linear mixed-model analysis, SPSS). ^$^Statistically significant vs. normal weight group (*p* < 0.05, linear mixed-model analysis, SPSS). **(G)** Pearson’s test of correlation between exercise-induced changes in leg press and glucose oxidation in myotubes. Δ = after exercise–before exercise. Full line represents the regression line for all donors (n = 18, Pearson’s correlation coefficient, r = 0.52, and *p* = 0.03), whereas stapled line represents the regression line for the overweight group (n = 11, Pearson’s correlation coefficient, r = 0.68, and *p* = 0.02).

Glucose metabolism in myotubes obtained from biopsies before and after 12 weeks of exercise is presented in Fig 2. Results for all participants combined (n = 18) are shown in Fig 2A–2C, and separated by BMI in Fig 2D–2F. We observed a 14% exercise-induced increase in fractional oxidation of glucose, but no exercise-induced effect on total cellular glucose uptake or oxidation for all participants (Fig 2C). When study group was separated by BMI, a significant exercise-induced increase in fractional glucose oxidation was observed in myotubes from the overweight group (Fig 2F), while total cellular glucose uptake and oxidation tended to be higher in the normal weight group compared to the overweight group after exercise (*p* = 0.07 and *p* = 0.06, respectively, Fig 2F). Furthermore, we found a significant correlation between exercise-induced improvement in maximal leg press and exercise-induced increase in glucose oxidation after exercise (Fig 2G, full line, r = 0.52, and *p* = 0.03), indicating a relationship between *in vivo* and *in vitro* findings that is not visible when only comparing before and after exercise. This correlation was also significant for the overweight group (Fig 2G, stapled line, r = 0.68, and *p* = 0.02). In myotubes established before exercise, oxidation and uptake of glucose were increased in the overweight group compared to the normal weight group (Fig 2D).

### No changes in AMPK phosphorylation in cultured human myotubes after 12 weeks of exercise

**Fig 3:**
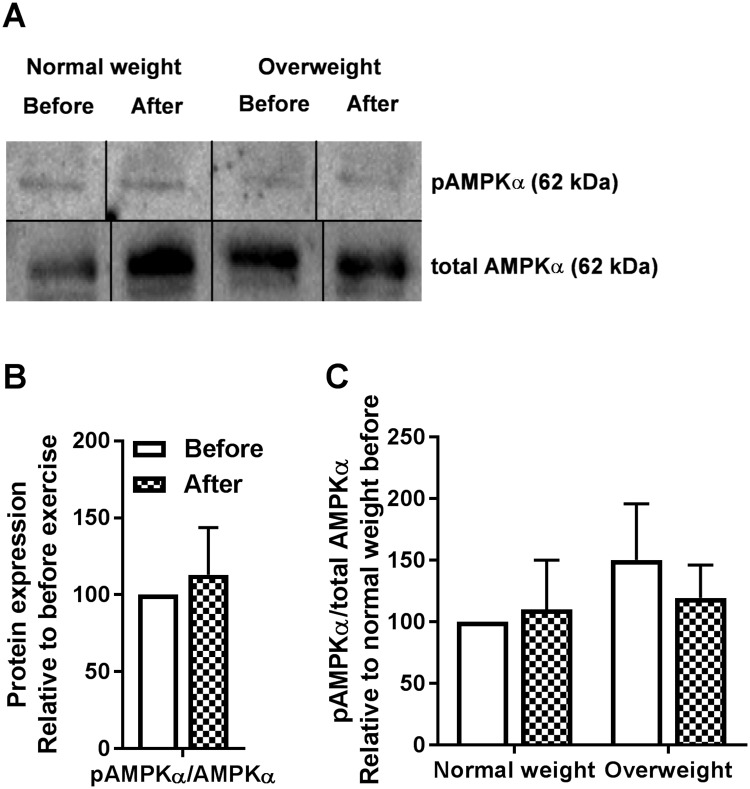
Effects of 12 weeks of exercise on myotube AMPKα phosphorylation. Satellite cells isolated from biopsies from *m*. *vastus lateralis* before and after 12 weeks of exercise were cultured and differentiated to myotubes. **(A-C)** AMPKα phosphorylation by immunoblotting. Protein was isolated and total AMPKα and pAMPKα expressions assessed by immunoblotting. A, one representative immunoblot. Bands selected from one membrane have been spliced together to show only relevant samples, as indicated by lines separating the spliced blots. B, quantified immunoblots for participants combined (n = 9) relative to before exercise. C, quantified immunoblots for study group when separated by BMI relative to normal weight before exercise (n = 5 in the normal weight group and n = 4 in the overweight group). Values are presented as means ± SEM. All samples were derived at the same time and processed in parallel.

AMPK plays an important role in cellular energy homeostasis, acting as a sensor of AMP/ATP or ADP/ATP ratios and thus cell energy level [35, 36]. To study whether AMPK could be a part of the observed exercise-induced changes on energy metabolism *in vitro* cultured myotubes was assessed by AMPKα (Thr172) phosphorylation (Fig 3). No changes in pAMPKα/total AMPKα ratio (Fig 3B) were observed in cells after exercise, nor between the two BMI groups (Fig 3C).

### No changes in mitochondria-related genes and proteins in cultured human myotubes after 12 weeks of exercise

**Fig 4:**
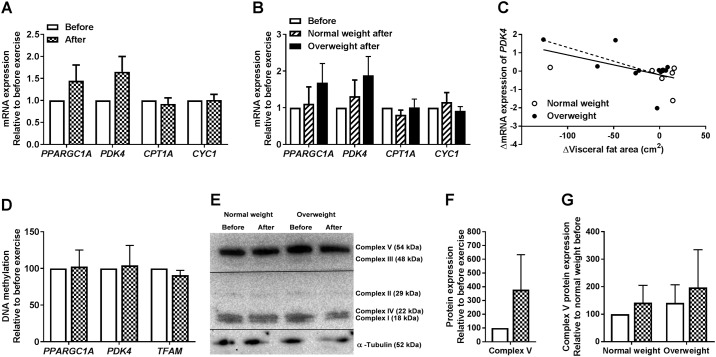
Effects of 12 weeks of exercise on mitochondria-related genes and proteins. Satellite cells isolated from biopsies from *m*. *vastus lateralis* before and after 12 weeks of exercise were cultured and differentiated to myotubes. **(A)** mRNA expression of *PPARGC1A*, *PDK4*, *CPT1A*, and *CYC1* after exercise relative to before exercise. mRNA was isolated and expression assessed by qPCR. All values were corrected for the housekeeping control *GAPDH*, and presented as means ± SEM for all participants combined (n = 18). **(B)** mRNA expression of *PPARGC1A*, *PDK4*, *CPT1A*, and *CYC1* after exercise relative to before exercise in study group when separated by BMI. mRNA was isolated and expression assessed by qPCR. All values were corrected for the housekeeping control *GAPDH*, and presented as means ± SEM (n = 7 in the normal weight group and n = 11 in the overweight group). **(C)** Pearson’s test of correlation was performed between exercise-induced changes in visceral fat area and mRNA expression of *PDK4* in myotubes. Δ = after exercise–before exercise. Full line represents the regression line for all donors (n = 18, Pearson’s correlation coefficient, r = -0.54, and *p* = 0.02), whereas stapled line represents the regression line for the overweight group (n = 11, Pearson’s correlation coefficient, r = -0.63, and *p* = 0.04). **(D)** DNA methylation of *PPARGC1A*, *PDK4* and *TFAM* after exercise relative to before exercise. gDNA was isolated and bisulfite treated, and methylation assessed by immunoblotting. Values are presented as means ± SEM (n = 6). **(E-G)** OXPHOS complexes by immunoblotting. Protein was isolated and OXPHOS complexes assessed by immunoblotting. E, one representative immunoblot. F, quantified immunoblots of complex V for participants combined. All values were corrected for the housekeeping control α-tubulin, and presented as means ± SEM (n = 10). G, quantified immunoblots of complex V in study group when separated by BMI. All values were corrected for the housekeeping control α-tubulin, and presented as means ± SEM (n = 5 in each group).

To study possible exercise-induced changes in oxidative capacity in the mitochondria we studied genes and proteins related to mitochondria (Fig 4). *PPARGC1A* codes for the master regulator of mitochondrial biogenesis PGC-1α [37–39], *PDK4*, *CPT1A* and *CYC1* are genes coding for proteins involved in metabolism in mitochondria [40–43], while *TFAM* codes for a mitochondrial transcription factor [44]. There were no significant exercise-induced changes in *PPARGC1A*, *PDK4* (p = 0.08), *CPT1A*, or *CYC1* for all participants combined (Fig 4A), nor when separated by BMI (Fig 4B). However, we observed a significant correlation between exercise-induced reduction in visceral fat area *in vivo* and increased mRNA expression of *PDK4* in the myotubes (Fig 4C, full line, *p* = 0.02, r = -0.54). This correlation was also significant for the overweight group (Fig 4C, stapled line, *p* = 0.04, r = -0.63). We also monitored DNA methylation of *PPARGC1A*, *PDK4* and *TFAM* genes in myotubes from a small subset of donors (n = 6, combination of both donor groups) before and after exercise (Fig 4D). Overall, there were no differences in CpG methylation within the regions we tested in *PPARGC1A*, *PDK4* or *TFAM*. However, 1 out of 8 CpGs tested in the *TFAM*-promoter was hypomethylated after exercise compared to before exercise (34% decrease, data not shown). Furthermore, we measured protein expression of the mitochondrial oxidative phosphorylation (OXPHOS) complexes (Fig 4E–4G), detected with an antibody cocktail recognizing complex I subunit NDUFB8, complex II subunit 30 kDa, complex III subunit Core 2, complex IV subunit II, and ATP synthase subunit alpha. Only complex V was quantifiable across the membranes. No clear exercise-induced changes were observed for participants combined (Fig 4F), nor when separated by BMI (Fig 4G).

### No change in genes related to lipid metabolism after 12 weeks of exercise in cultured human myotubes

**Fig 5:**
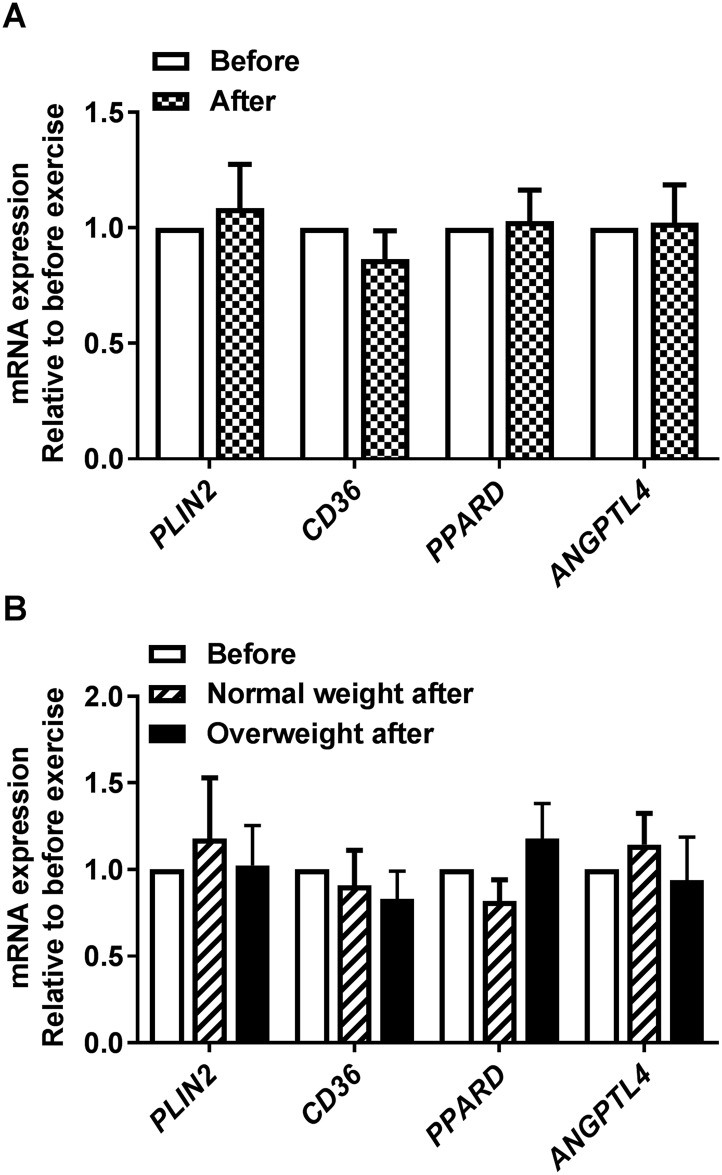
Effects of 12 weeks of exercise on myotube expression of lipid metabolism associated genes. Satellite cells isolated from biopsies from *m*. *vastus lateralis* before and after 12 weeks of exercise were cultured and differentiated to myotubes. mRNA was isolated and expression assessed by qPCR. **(A)** mRNA expression after exercise relative to before exercise for all participants combined. All values were corrected for the housekeeping control *GAPDH*, and presented as means ± SEM (n = 18). **(B)** mRNA expression after exercise relative to before exercise for study group when separated by BMI. All values were corrected for the housekeeping control *GAPDH*, and presented as means ± SEM (n = 7 in the normal weight group and n = 11 in the overweight group).

Some genes related to lipid metabolism were also examined to further probe mechanisms behind the exercise-induced metabolic changes observed *in vitro*. mRNA of *PLIN2*, involved in coating of lipid droplets and thus lipid accumulation [45, 46], was not significantly different after the exercise intervention for all participants (Fig 5A) or when the study group was separated by BMI (Fig 5B). Neither was mRNA of *CD36*, an important transporter of fatty acids across the plasma membrane [47, 48] (Fig 5A and 5B). We have previously shown that activation of PPARδ increased lipid oxidation in human skeletal muscle cells [49]. Gene expression of *PPARD* or the PPAR-target gene *ANGPTL4* [50–52] also showed no exercise-induced changes (Fig 5A), nor when study group was separated by BMI (Fig 5B). We also monitored DNA methylation of *PPARD* in the small subset of donors (n = 6, combination of both donor groups) before and after exercise, but no differences in CpG methylation within the region we tested were observed (data not shown).

### No changes in insulin response in cultured human myotubes after 12 weeks of exercise

**Fig 6:**
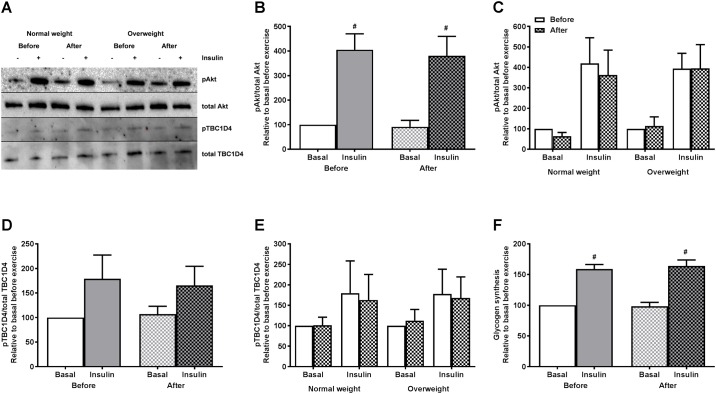
Effects of 12 weeks of exercise on myotube Akt phosphorylation, TBC1D4 phosphorylation and glycogen synthesis with or without 100 nmol/l insulin. Satellite cells isolated from biopsies from *m*. *vastus lateralis* before and after 12 weeks of exercise were cultured and differentiated to myotubes. **(A-C)** Akt phosphorylation by immunoblotting. Protein was isolated and total Akt and pAkt expressions assessed by immunoblotting. A, one representative immunoblot. B, quantified immunoblots relative to basal before exercise for participants combined. Values are presented as means ± SEM (n = 9). C, quantified immunoblots relative to basal before exercise for study group when separated by BMI (n = 4 in the normal weight group and n = 5 in the overweight group). **(A, D and E)** TBC1D4 phosphorylation by immunoblotting. Protein was isolated and total TBC1D4 and pTBC1D4 expressions assessed by immunoblotting. A, one representative immunoblot. D, quantified immunoblots relative to basal before exercise for participants combined. Values are presented as means ± SEM (n = 10). E, quantified immunoblots relative to basal before exercise for study group when separated by BMI (n = 5 in both groups). All samples were derived at the same time and processed in parallel. **(F)** Glycogen synthesis relative to basal before exercise. Values are presented as means ± SEM (n = 5). Absolute values (range) representing 100%: Basal glycogen synthesis 3.9–15.4 nmol/mg protein. ^#^Statistically significant vs. basal before exercise (*p* < 0.05, paired t test).

Both donor groups experienced increased GIR after exercise (Table 2). To examine whether the improved insulin sensitivity *in vivo* was mirrored *in vitro* in the myotubes, the response to 100 nmol/l insulin was assessed by measurement of Akt (Ser473) phosphorylation, TBC1D4 (Thr642) phosphorylation, IRS1 (Tyr612) phosphorylation, and glycogen synthesis (Fig 6). No changes in the basal level of pAkt/total Akt ratio or pTBC1D4/total TBC1D4 ratio were observed in cells after exercise. As expected, insulin significantly increased the pAkt/total Akt ratio in myotubes from both groups before and after exercise (Fig 6A and 6B), whereas there were no significant effect of insulin on pTBC1D4/total TBC1D4 ratio (Fig 6A and 6D). When the study group was separated by BMI, no significant differences in basal or insulin-stimulated levels of pAkt/total Akt ratio or pTBC1D4/total TBC1D4 ratio were observed (Fig 6C and 6E, respectively). No changes in the basal level or insulin-stimulated levels of pIRS1/total IRS1 ratio were observed (data not shown). Furthermore, no changes in the basal level of glycogen synthesis were observed in myotubes, and insulin significantly increased glycogen synthesis by about 1.5-fold both before and after exercise (Fig 6F). Thus, there was no exercise-effect on insulin-stimulated Akt phosphorylation, TBC1D4 phosphorylation or glycogen synthesis.

### Decreased *IRS1* mRNA expression and increased DNA methylation within first exon region of *IRS1* after 12 weeks of exercise in cultured human myotubes

**Fig 7:**
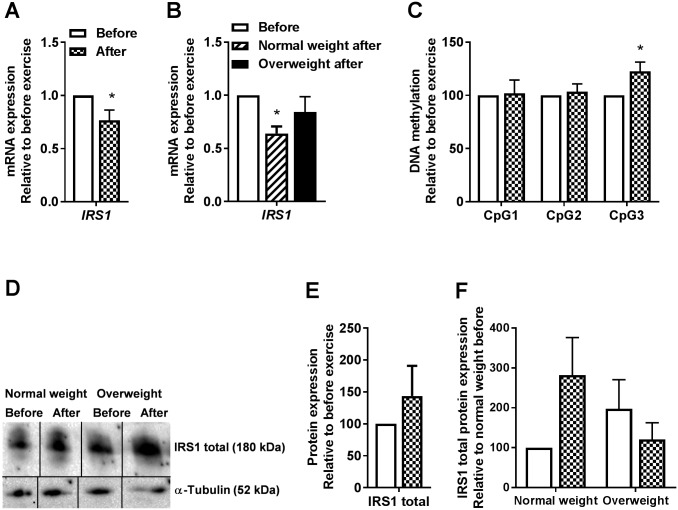
Effects of 12 weeks of exercise on myotube *IRS1* gene expression and *IRS1* first exon DNA methylation. **(A)** *IRS1* mRNA expression after exercise relative to before exercise for participants combined. mRNA was isolated and expression assessed by qPCR. All values were corrected for the housekeeping control *GAPDH*, and presented as means ± SEM (n = 8). *Statistically significant vs. before exercise (*p* < 0.05, paired t test). **(B)** *IRS1* mRNA expression after exercise relative to before exercise for study group when separated by BMI. mRNA was isolated and expression assessed by qPCR. All values were corrected for the housekeeping control *GAPDH*, and presented as means ± SEM (n = 3 in the normal weight group and n = 5 in the overweight group). *Statistically significant vs. before exercise (*p* < 0.05, paired t test). **(C)** *IRS1* first exon DNA methylation after exercise relative to before exercise. gDNA was isolated and bisulfite treated, and methylation was assessed by pyrosequencing. Values are presented as means ± SEM (n = 6). *Statistically significant vs. before exercise (*p* < 0.05, paired t test). **(D-F)** IRS1 total protein expression. Protein was isolated and total IRS1 expression assessed by immunoblotting. D, one representative immunoblot. Bands selected from one membrane have been spliced together to show only relevant samples, as indicated by lines separating the spliced blots. E, quantified immunoblots relative to before exercise for participants combined. All values were corrected for the housekeeping control α-tubulin, and presented as means ± SEM (n = 9). G, quantified immunoblots relative to before exercise for study group when separated by BMI. All values were corrected for the housekeeping control α-tubulin, and presented as means ± SEM (n = 5 in the normal weight group and n = 4 in the overweight group). All samples were derived at the same time and processed in parallel.

To further study the insulin signaling pathway, we also measured mRNA expression, DNA methylation and protein expression of IRS1 (Fig 7). We found that the mRNA expression of *IRS1* was significantly decreased by 31% after exercise (n = 8, Fig 7A), which was only significant in myotubes from the normal weight group upon separation by BMI (n = 3 in the normal weight group and n = 5 in the overweight group, Fig 7B). Furthermore, DNA methylation of 1 out of 3 CpGs tested within the first exon region of *IRS1* was significantly increased by 23% (n = 6, Fig 7C). There were no exercise-induced changes in protein expression of IRS1 detected with immunoblotting (n = 9, Fig 7E), nor when study group was separated by BMI (n = 5 in the normal weight group and n = 4 in the overweight group, Fig 7F).

## Discussion

We show that 12 weeks of exercise alters metabolism and gene expression of cultured human myotubes. Fatty acid metabolism and fractional glucose oxidation were significantly increased in myotubes established from skeletal muscle isolated from sedentary men after 12 weeks of exercise. These exercise-induced metabolic changes in fatty acid metabolism in myotubes were more predominant in cells from overweight subjects. Moreover, we observed a significant exercise-induced decrease in mRNA expression of *IRS1*, as well as DNA hypermethylation in the first exon of *IRS1*, however not detectable on protein level.

Bourlier *et al*. showed that cultured myotubes retained the exercise-trained phenotype *in vitro* concerning some aspects of glucose metabolism [16]. Their study involved 8 weeks of aerobic exercise intervention and included only obese individuals [16]. In the present study we examined a broader group of subjects including normal weight and overweight men, a longer exercise intervention as well as a combination of aerobic and anaerobic exercise, to observe and possibly explain differences in energy metabolism in cultured myotubes *in vitro* after the *in vivo* exercise intervention, and also to explore whether BMI of the subjects affected the results.

As expected, the exercise intervention significantly increased VO_2max_ (overweight group), chest press, cable pull-down, and leg press capacity. The exercise intervention also improved the metabolic health, with a significant increase in GIR, as well as a small, but significant reduction in BMI. VO_2max_ was not significantly increased in the normal weight group (*p* = 0.053) even though they complied to the exercise intervention equally well [26]. The mean increase was variable between the participants, and combined with the smaller sample size it may explain the lack of statistical difference.

With the combination of aerobic and anaerobic exercises and longer intervention we have several interesting findings with regard to fatty acid metabolism in myotubes established from biopsies taken before and after 12 weeks of exercise. We observed a significantly increased oleic acid oxidation, fractional oxidation and lipid accumulation in the cells, statistically significant only in the overweight group (except total cellular oleic acid uptake).

In our study there are no data on lipid utilization *in vivo* or *ex vivo* to directly compare with *in vitro* data. However, from the same clinical study muscle lipid content, measured by magnetic resonance spectrometry *in vivo* and electron microscopy *ex vivo*, was found to be significantly reduced after the exercise intervention [26, 28], in line with an increase in lipid metabolism *in vitro*.

An exercise-induced increase in lipid oxidation in cultured myotubes is also in accordance with findings from others in skeletal muscle *in vivo* during and after combined types of exercise [7, 53]. A study by Ramos-Jiménez *et al*. [8] showed that lipid oxidation was increased in endurance trained men (athletes trained at a competitive level) compared to untrained men, as measured by lower respiratory exchange ratio. Increased lipid oxidation after exercise is also in line with observations from an *in vitro* model (electrical pulse stimulation) of myotube exercise [54, 55]. Bourlier *et al*. did not observe exercise-induced differences in lipid metabolism in cultured myotubes, however, they hypothesized that longer exercise interventions and/or interventions including different types of exercise might lead to functional changes in lipid metabolism [16].

Bourlier *et al*. [16] reported increased glucose metabolism in myotubes from obese subjects after an 8-week aerobic exercise intervention. In our study we observed increased fractional oxidation of glucose, statistically significant only in the overweight group, as well as a significant correlation between exercise-induced increased maximal leg press capacity and increased oxidation of glucose in the cells, indicating a relationship between glucose oxidation and exercise outcome. However, the effects of exercise on glucose metabolism were less pronounced in our study than described by Bourlier *et al*. [16], possibly explained by different donor groups and exercise programs. Increased storage of glycogen is a well-reported physiologic response to exercise as a mean to increase endurance capacity during submaximal exercise [11, 56], and Bourlier *et al*. also reported increased basal glycogen synthesis in myotubes cultured from satellite cells after exercise *in vivo* [16]. However, this was not observed in this study, possibly caused by different study conditions.

In this study we have compared myotubes from normal weight and overweight subjects. In pre-training myotubes we found increased oxidation and uptake of glucose and lower lipid accumulation in the overweight group compared to the normal weight group, as well as a possible association between lipid accumulation *in vitro* and insulin sensitivity *in vivo*. Several previous studies show no significant donor-related differences i basal glucose oxidation in myotubes [54, 57–59], however Gaster [17] observed increased glucose oxidation in myotubes from obese patients with T2D compared to myotubes from lean donors. It was suggested that under certain conditions metabolism of myotubes from diabetic donors relies more on glucose oxidation than myotubes from lean donors [17]. We have previously reported lower lipid accumulation in myotubes from obese subjects with T2D compared to myotubes from obese non-diabetic donors, explained by a reduced capacity for lipid accumulation and increased lipolysis [60]. Our overweight donors are not diabetic, however this donor group had reduced pre-training insulin sensitivity and myotubes from this group may resemble cells from T2D donors in some ways. The donor-dependent differences in glucose metabolism and lipid accumulation found in pre-training myotubes were evened out after exercise, in line with the increased response to exercise in myotubes from the overweight group.

Satellite cells are usually dormant *in vivo* until they are challenged with growth or injury [13], *e*.*g*. exercise. We observed changes in energy metabolism in skeletal muscle cells following exercise intervention, and aimed to determine whether gene or protein expression were coincident with the observed changes in energy metabolism.

Despite the increased fatty acid oxidation, we did not observe any significant exercise-induced differences in phosphorylation of AMPKα, and no changes in mRNA expression levels of mitochondria-related genes or genes related to fatty acid metabolism. However, there was a significant correlation between reduced visceral fat area *in vivo* and higher mRNA expression of *PDK4 in vitro*. *PDK4* is involved in phosphorylation and inactivation of the pyruvate dehydrogenase complex (PDC). Increased expression of *PDK4* inhibits PDC and reduces glucose oxidation, which makes *PDK4* a major regulatory metabolic enzyme in skeletal muscle as it is involved in switching from carbohydrate to lipid utilization [41, 61, 62]. Bourlier *et al*. [16] found a reduced *PDK4* mRNA expression after exercise in cultured myotubes, in line with the increased glucose oxidation [16], while we previously have found that increased lipid oxidation of cultured human myotubes *in vitro* simultaneously also increased *PDK4* expression [49, 55, 63]. Thus, the correlation between reduced visceral fat area and increased *PDK4* expression may indicate a relationship between lipid metabolism *in vivo* and *in vitro*.

DNA methylation has been proposed as a molecular mechanism for exercise-mediated changes in metabolic health [15] and has been associated with transcriptional silencing [64], possibly by blocking the promoter that activating transcription factors normally bind. In our study, DNA methylation of the mitochondrial genes *TFAM* and *PDK4* were not changed in myotubes after exercise. This is in contrast to findings *ex vivo* after acute exercise. Barrès *et al*. [65] showed that acute exercise increased mRNA expression of *PDK4* and *PPARGC1A* in skeletal muscle, and that changes in methylation was part of the explanation. However, we found both hypermethylation of *IRS1* and reduction of *IRS1* mRNA expression in cultured myotubes after 12 weeks of training, whereas protein expression apparently was unchanged. The functional significance of these findings is unknown and not easy to explain. Protein expression of IRS1 has previously been shown to be both increased [66] and decreased [67] in human skeletal muscle after exercise. We have recently shown enhanced tyrosine phosphorylation of IRS1, concomitant with increased glucose metabolism in cultured myotubes obtained from donors before and after gastric by-pass surgery [68]. Our study indicates that exercise-induced changes in promoter methylation may be retained in satellite cells and during transition of these precursor cells to myoblasts and finally to myotubes, however, at present we cannot explain a possible link between this and the metabolic changes observed.

Disturbances in energy metabolism of skeletal muscle are associated with metabolic diseases related to insulin resistance [69, 70]. *In vivo* we found a significant increased GIR after training i both donor groups, indicating increased insulin sensitivity, while no exercise-induced changes in *in vitro* insulin response (*i*.*e*. insulin-stimulated Akt phosphorylation, TBC1D4 phosphorylation or glycogen synthesis) were observed. This could be explained by sub-optimal experimental conditions (*i*.*e*. a maximal insulin stimulation), though we have previously been able to detect donor-specific differences in insulin-response with the same experimental setup [20, 60]. We hypothesize therefore that the lack of these effects are a result of the underlying study *in vivo* where the two donor groups were quite similar with regard to insulin sensitivity, and that the difference were too small to be able to detect *in vitro*.

In conclusion, our data show that a combination of aerobic and anaerobic exercise mediates changes in fatty acid and glucose metabolism in skeletal muscle cells. Thus, certain impacts of exercise *in vivo* are retained in myotubes established from satellite cells, and our findings may indicate that cultured, passaged myoblasts established from these progenitor cells and differentiated into myotubes, can be used as a model system for studying mechanisms related to exercise and metabolic diseases. Furthermore, we observed that the exercise-induced changes were predominant in the overweight group. Future studies are required to explore whether epigenetic or other changes can explain this relationship further, and to get a deeper insight into molecular mechanisms behind changes in energy metabolism in myotubes after an exercise intervention.

## Supporting information

S1 Fig No differences in protein expression of differentiation markers. Satellite cells isolated from biopsies from *m*. *vastus lateralis* before and after 12 weeks of exercise were cultured and differentiated to myotubes. **(A, B)** MHCI expression by immunoblotting. Protein was isolated and MHCI expression assessed by immunoblotting. A, one representative immunoblot. Bands selected from one membrane have been spliced together to show only relevant samples, as indicated by lines separating the spliced blots. B, quantified immunoblots for study group when separated by BMI relative to normal weight before exercise (n = 5 in both groups). **(A, C)** MHCIIa expression by immunoblotting. Protein was isolated and MHCIIa expression assessed by immunoblotting. A, one representative immunoblot. Bands selected from one membrane have been spliced together to show only relevant samples, as indicated by lines separating the spliced blots. C, quantified immunoblots for study group when separated by BMI relative to normal weight before exercise (n = 5 in both groups). All values were corrected for the housekeeping control α-tubulin. Values are presented as means ± SEM. All samples were derived at the same time and processed in parallel.
(TIF)

## Abbreviations

AMPK: AMP-activated protein kinase
ANGPTL4: angiopoietin-like 4
BMI: body mass index
CA: cell-associated radioactivity
CD36: fatty acid translocase
CPT1A: carnitine palmitoyltransferase 1A
CYC1: cytochrome c-1
GAPDH: glyceraldehyde 3-phosphate dehydrogenase
GIR: glucose infusion rate
IRS1: insulin receptor substrate 1
MHC: myosin heavy chain
OXPHOS: oxidative phosphorylation
PDK4: pyruvate dehydrogenase kinase, isoenzyme 4
PPARGC1A: peroxisome proliferator-activated receptor gamma, coactivator 1 alpha
PLIN2: perilipin 2
PPARD: peroxisome proliferator-activated receptor delta
RPLP0: large ribosomal protein P0
T2D: type 2 diabetes mellitus
TBC1D4: TBC1 domain family member 4
TFAM: transcription factor A, mitochondrial
VO_2max_: maximal oxygen uptake
