# Supplementary figures and images for: Exercise in vivo marks human myotubes in vitro: Training-induced increase in lipid metabolism
Source: PLoS One. 2017 Apr 12;12(4):e0175441. doi: 10.1371/journal.pone.0175441 (PMC5389842; doi:10.1371/journal.pone.0175441)

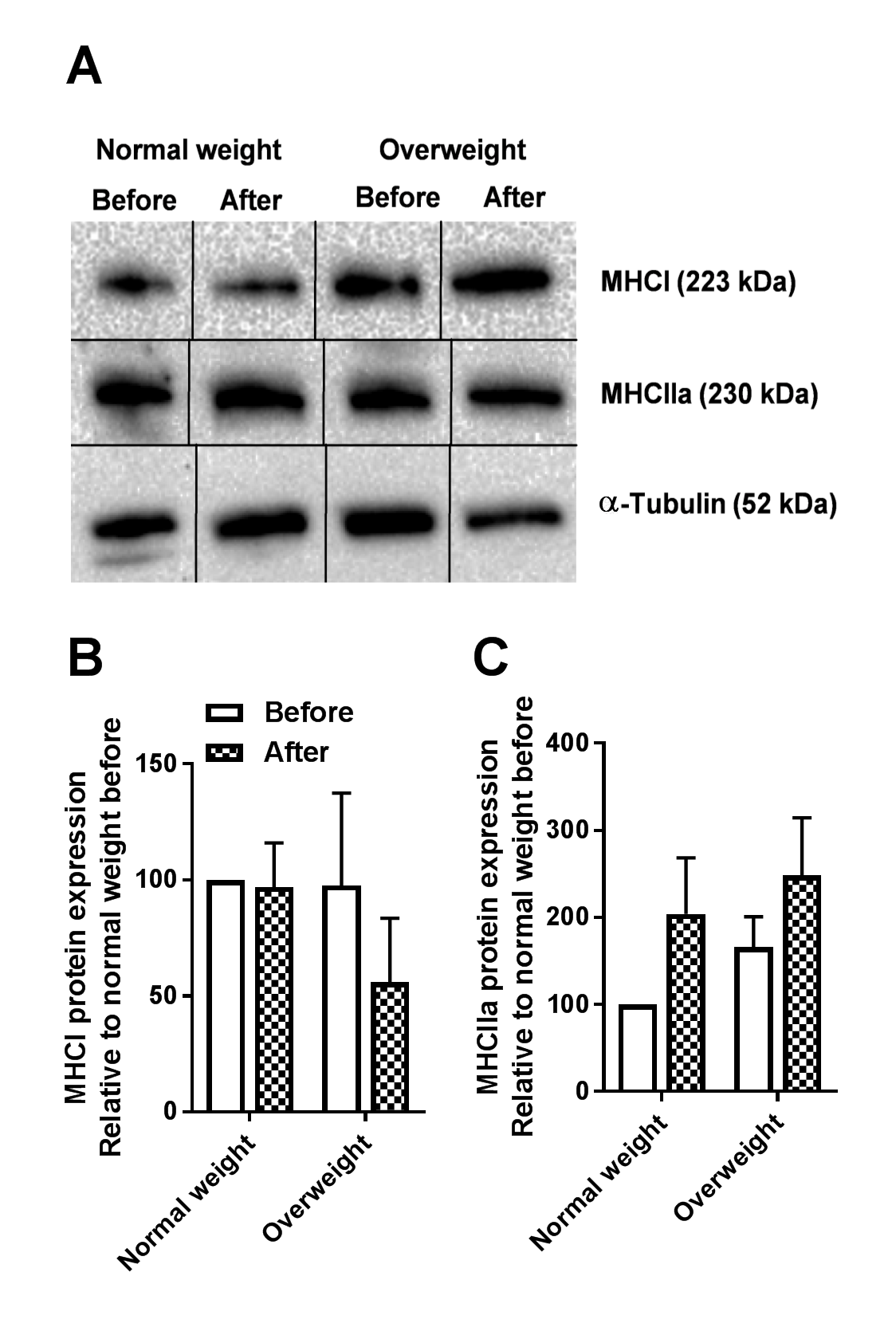

Supplement: S1 Fig — Satellite cells isolated from biopsies from m. vastus lateralis before and after 12 weeks of exercise were cultured and differentiated to myotubes. (A, B) MHCI expression by immunoblotting. Protein was isolated and MHCI expression assessed by immunoblotting. A, one representative immunoblot. Bands selected from one membrane have been spliced together to show only relevant samples, as indicated by lines separating the spliced blots. B, quantified immunoblots for study group when separated by BMI relative to normal weight before exercise (n = 5 in both groups). (A, C) MHCIIa expression by immunoblotting. Protein was isolated and MHCIIa expression assessed by immunoblotting. A, one representative immunoblot. Bands selected from one membrane have been spliced together to show only relevant samples, as indicated by lines separating the spliced blots. C, quantified immunoblots for study group when separated by BMI relative to normal weight before exercise (n = 5 in both groups). All values were corrected for the housekeeping control α-tubulin. Values are presented as means ± SEM. All samples were derived at the same time and processed in parallel. (TIF) [file pone.0175441.s001.tif]
